# Cerebrospinal fluid apoe glycosylation associates with biomarkers of Alzheimer’s Disease pathology

**DOI:** 10.1002/alz.091438

**Published:** 2025-01-09

**Authors:** Dobrin Nedelkov, Zoe Tsokolas, Isabel Sible, Wendy J Mack, S Duke Han, John CS Rodman, Hussein N Yassine

**Affiliations:** ^1^ Isoformix Inc., Sugar Land, TX USA; ^2^ University of Southern California, Los Angeles, CA USA

## Abstract

**Background:**

The apolipoprotein E (ApoE) Ɛ4 allele is associated with a significant risk for both late‐onset Alzheimer’s Disease (AD) development and cerebral amyloidosis, but the degree to which cerebrospinal fluid (CSF) apoE glycosylation affects disease progression is unclear. The objective of this study was to examine the relationship of CSF apoE glycosylation with t‐tau, p‐tau181, and Aβ1‐42 CSF levels, and to delineate the effect of the APOE4+ genotype (vs E4‐) on glycosylation.

**Method:**

Total glycosylation and apoE isoform‐specific glycosylation were analyzed in baseline plasma and CSF samples from a longitudinal cohort of older individuals (n=188, ages 55 ‐ 89) from the Alzheimer’s Disease Neuroimaging Initiative (ADNI). ApoE glycosylation was assayed using Mass Spectrometric Immunoassay (MSIA) that simultaneously detects the apoE isoforms and their glycosylation percentage. We performed pairwise comparisons between each apoE isoform and utilized linear mixed‐effects modeling to analyze longitudinal associations of apoE glycosylation with AD biomarkers, adjusting for age, sex, race, education, and clinical status.

**Result:**

We examined glycosylation as a function of individual apoE isoforms and found that both total and secondary CSF glycosylation had the same trend: the E4 isoform had an overall lower % glycosylation compared to E2 and E3 isoforms (p<0.0001). Similarly, the E3 isoform had lower total and secondary CSF % glycosylation compared to the E2 isoform (p<0.0001). We further assessed whether the % apoE glycosylation in the baseline samples were associated with CSF t‐tau, p‐tau181, and Aβ1‐42 CSF levels in the longitudinal samples. Total CSF and secondary CSF glycosylation demonstrated highly significant associations with decreased levels of t‐tau (p < 0.002; p < 0.02 respectively) and p‐Tau181 (p< 0.006; p<0.02 respectively) over a 10‐year period. Secondary CSF glycosylation was significantly positively correlated with increased CSF Aβ1‐42 levels over a 10‐year period (p < 0.04).

**Conclusion:**

The degree of CSF ApoE glycosylation is strongly associated with concentrations of CSF t‐tau, p‐Tau181, and Aβ1‐42, and is predictive of biomarker changes over time. These novel discoveries underscore the importance of understanding the mechanisms involved in apoE glycosylation in the brain, which can lead to new targets and therapies for AD.

